# Red blood cell glutathione peroxidase activity in female nulligravid and pregnant rats

**DOI:** 10.1186/1477-7827-7-7

**Published:** 2009-01-26

**Authors:** Giuseppe Gallo, Guglielmo Martino

**Affiliations:** 1 Department of Cell Biology, University of Calabria, Rende (CS), Italy

## Abstract

**Background:**

The alterations of the glutathione peroxidase enzyme complex system occur in physiological conditions such as aging and oxidative stress consequent to strenuous exercise.

**Methods:**

Authors optimize the spectrophotometric method to measure glutathione peroxidase activity in rat red blood cell membranes.

**Results:**

The optimization, when applied to age paired rats, both nulligravid and pregnant, shows that pregnancy induces, at seventeen d of pregnancy, an increase of both reactive oxygen substance concentration in red blood cells and membrane glutathione peroxidase activity.

**Conclusion:**

The glutathione peroxidase increase in erythrocyte membranes is induced by systemic oxidative stress long lasting rat pregnancy.

## Background

The aim of the present research is to evaluate the contribution of the enzymatic antioxidant glutathione peroxidase (GP) by optimization of the spectrophotometric method of Paglia and Valentine [1] so that it can be applied to blood samples from several animals differing from sex, age and species. After evaluating the usefulness of the method, it was thus, applied to study the effect of the different levels of oxidative stress consequent to pregnancy.

GP (that is PDB 1GP1, according to Protein Data Bank, and EC 1.11.1.9, according to the Enzyme Commission numbering) is the main term of an enzyme family with peroxidase activity. GP, discovered in 1957 by Mills [2], has the function to reduce lipid hydroperoxides to their corresponding alcohols and to reduce free hydrogen peroxide in water. GP1 is found in the cytoplasm of nearly all mammalian tissues, whose preferred substrate is hydrogen peroxide. The GP reaction is:

2GSH + H_2_O_2 _→ GS-SG + 2H_2_O

where GSH represents reduced monomeric glutathione, and GS-SG represents glutathione disulfide. Glutathione reductase (GR) then reduces the oxidized glutathione to complete the cycle:

GS-SG + NADPH + H^+ ^→ 2 GSH + NADP^+^.

**Figure 1:**
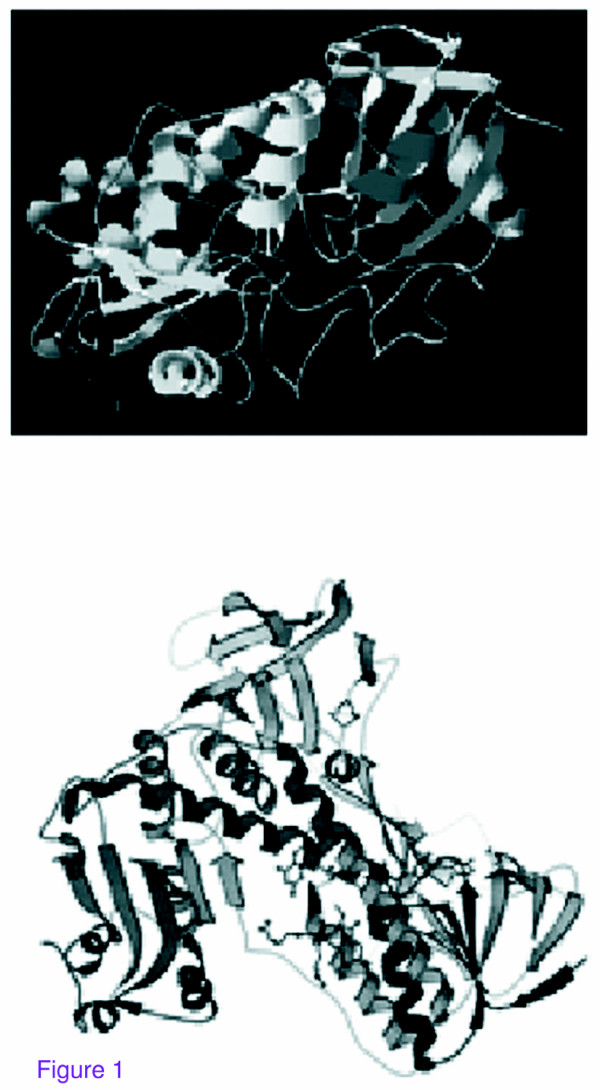
**3D view of GP-GR structure (from NCBI PDB)**.

GP is a selenium-containing glycoprotein. (fig. 1). As the integrity of subcellular membranes depends heavily on GP, which in turn depends on selenium, the mechanism of GP is at the Selenocystein site, in a Se^- ^form at resting state. This is oxidized by the peroxide to SeOH subsequently trapped by a GSH molecule to give Se-SG and by another GSH molecule to Se^- ^again, releasing a GS-SG by-product. The human GR, also known as GSR, is an enzyme. (E.C. 1.8.1.7) it reduces glutathione disulfide (GSSG) to the sulphydryl form GSH, which is an important cellular antioxidant. (fig. 1). For each mol of oxidized glutathione (GSSG) one mol of NADPH is required to produce GSH. NADPH reduces FAD present in GSR to produce a transient FADH^- ^anion. This anion then quickly breaks a disulfide bond and leads to Cys_63 _nucleophilically attacking the nearest sulfide unit in the GSSG molecule (promoted by His_467_) thus creating a mixed disulfide bond (GS-Cys_58_) and a GS^- ^anion. His_467 _of GSR then protonates the GS^- ^to form the first GSH. Afterwards, Cys_63 _nucleophilically attacks this sulfide releasing a GS^- ^anion, thereby creating the second GSH. So, for each GSSG two reduced GSH antioxidant molecules are produced, scavenging reactive oxygen species in the cell. In cells exposed to high levels of oxidative stress, like red blood cells (RBC), up to 10% of the glucose consumption may be directed to the pentose phosphate pathway (PPP) for the production of the NADPH needed for this reaction. In erythrocytes, if the PPP does not function, then the (RBC) oxidative stress will lead to lysis, anemia.

**Figure 2:**
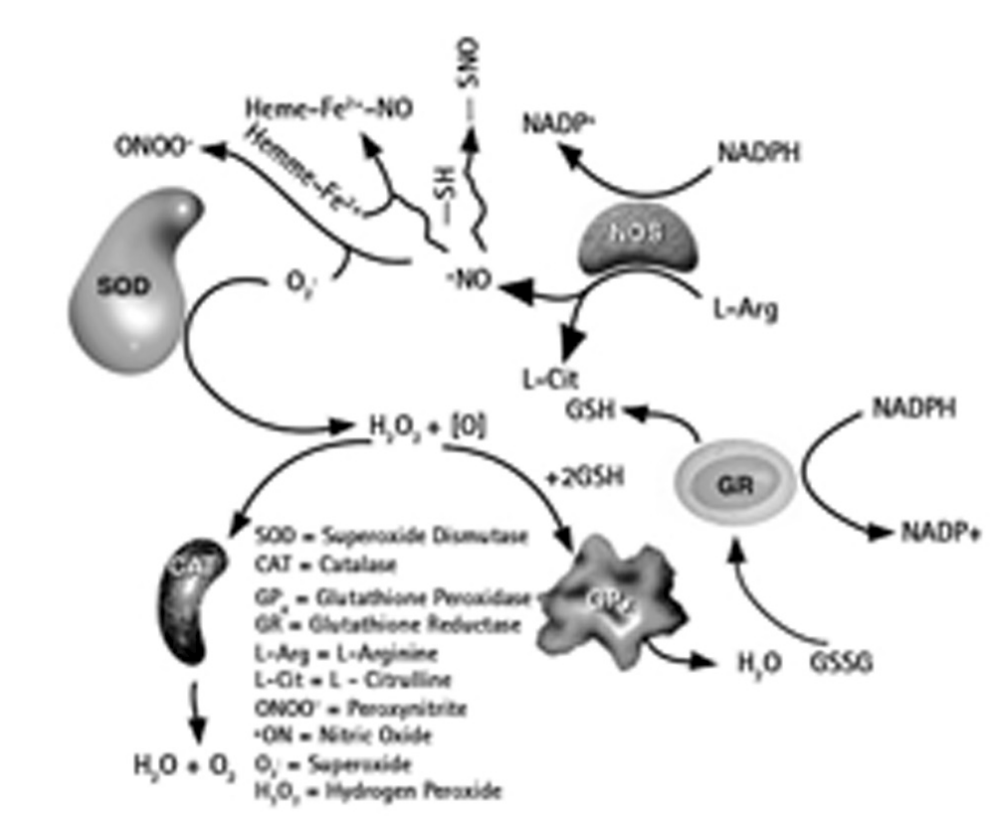
**Interaction of GP with the oxidative stress (Merck, Calbiochem 2006/2007 catalogue)**.

Alterations of this current production of peroxides and free radicals damage structural components of the cell. Reactive oxygen species can be beneficial, as against pathogens, and are also used in cell signalling. Oxidative stress (formulated in Harman's free radical theory of aging) is also thought to contribute to the process. Recent evidence suggests that oxidative stress may also promote life expectancy [3,4]. Recent findings in male support the process and suggest that antioxidants may modulate disease prevalence in humans. The interactions with the oxidative stress are resumed in figure 2.

At present, there is not sufficient information on oxidative stress and pregnancy available in literature. Ara et al. [5] studied peritoneal adhesions in rats. Jackson et al. [6] evaluated the association between oxidative stress and endometriosis and found only a weak association between thiobarbituric acid-reactive substances (nmol/ml) in serum and endometriosis in 10 pregnant females on 32 total studied. Vanderlelie and Perkins [7] evaluated the oxidative stress at the end of the reproduction in rats in placental and liver tissues, but they do not describe the alteration of GP activity, resulting from the coordination of tissue GSH reductase and GSSG peroxidase enzyme activities in both selenium deficiency and L-NAME oral administration.

The authors verify that GP in RBCs membranes works as a complex to show the comprehensive systemic effect of using GSH to scavenge ROS in RBCs, producing GSSG and the correspondent hydroperoxid equivalent to ROS. Only if all NADPH is oxidized used to replenish GSH stores in the membrane preparations, than the determination of NADPH oxidized by GP, represents a true measurement of the specific enzyme complex [8,9].

Concerning the hypertension mechanisms, the oxidative stress was shown in the rostral ventrolateral medulla (RVLM) [10] and in kidney [11] and are both present in spontaneously hypertensive rats (SHR). Zhou [12] demonstrated that oxidative stress in pregnancy can increase, even if the exact either pro-oxidant or antioxidant status in pregnancy-induced hypertensive patients is not clear [13]. There is no study on rat blood and on the relationship between hypertension and pregnancy.

## Methods

Sixteen weeks old nulligravid female Wistar rats were housed at a constant temperature (22°C) in a 12 hours light and dark cycle environment with free access to food and drinking water. Animals were randomised in two groups (n = 6) and fed a standard diet (MilRatti Stefano Morini, S. Polo D'Enza (RE)). The animals were treated according to the european community prescriptions [EU (86/609/EEC)], then cycled and mated with fertile males at proestrous on 120 d of age, with a positive vaginal smear for sperm, the day after proestrous indicating d 0 of pregnancy. The remaining animals continued on their diets as nulligravid controls. On d 140 pregnant rats were submitted to blood sampling under ether (50 g/kg body weight) anesthesia according to the european community prescriptions [EU (86/609/EEC)] on animal care.

### Processing of tissue samples and hemoglobin estimation

Blood, 100 μL of sample drawn by cardiac puncture under ether anesthesia (50 g/kg body weight) are centrifuged and washed twice with 5 mL of 0,9% NaCl. Isolated RBCs are hemolyzed by addition of 1 mL of distilled H_2_O. Hemoglobin concentration is determined by mixing 1 mL of Hemoglobin test (Sclavo diagnostics, Siena, Italy) with 0,1 mL of hemolysate. The absorbance at 546 nm is measured in a Shimadzu UVPC 2100 spectrophotometer (A_546 _× 16 = mg Hb/mL) against a blank containing water instead of hemolysate. The hemolysate is exactly diluted to 3 mg of Hb per millilitre. From this solution, 1 mL is mixed with 0.5 mL of transformation solution (4.5 mM KCN and 0.45 mM K_3 _[Fe(CN)_6_] adjusted with 0.25 M potassium dihydrogen phosphate to pH 7.0). After 5 min, transformation to cyanmethemoglobin is complete at room temperature.

### Glutathione peroxidase assay

GP activity was determined by a modified method of Paglia and Valentine (1967) [1] Activity was determined spectrophotometrically by coupling the oxidation of glutathione and NADPH using GR. Briefly, 1 mL of assay mixture contains optimized concentrations of the following chemicals: 0,5 M K_2_HPO_4 _(pH 7.0), 2,5 mM EDTA, 0,18 U/mL GR, 100 mM glutathione and 10 mM reduced NADPH and tissue extract (0,5 mL) was added in the spectrophotometer cuvette along with 0,1 mL of 60 mM cumene hydroperoxide, a suitable substrate for GP.

**Figure 3:**
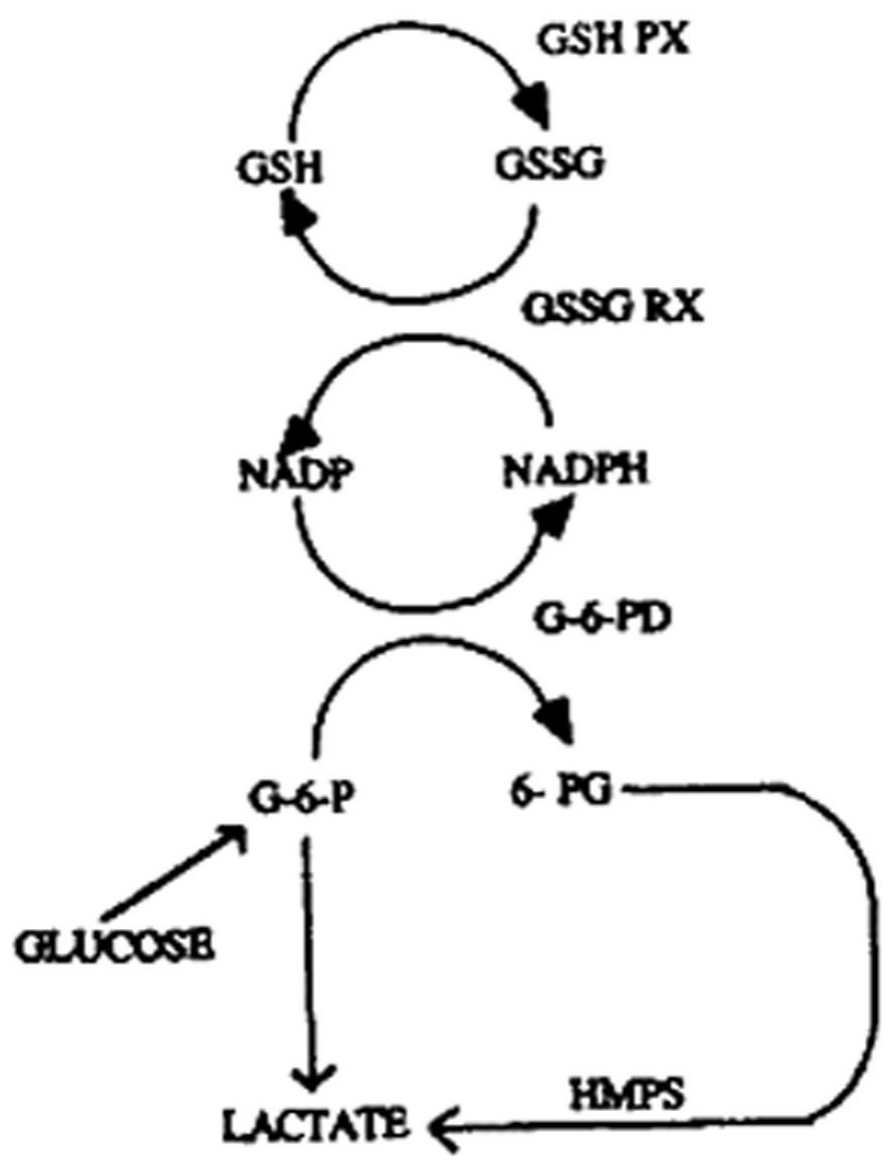
**Reciprocal relationships among GP (= GSH PX), GR (= GSSG RX) and G-6-PD (= glucose-6-phosphate dehydrogenase) enzyme activities (18)**.

The mixture was placed into a 1 mL cuvette and read with Shimadzu UVPC 2100 spectrophotometer set at 366 nm at 37°C. The method optimization takes into account the functional data of GP complex:

1) the peroxilipid is reduced by GSH (reduced glutathione) to hydroxilipids (GP activity);

2) GSH is oxidized to GSSG (oxidized glutathione) (GP);

3) GSSG is reduced to GSH by NADPH (GR) as resumed in figure 3.

According to the cited figure 3[14], the increase of NADPH concentration from 2,5 mM [1] up to 10 mM also increases the velocity of GR and the GSH reserve levels, that are increased from 10 mM up to 100 mM, in such a way to always saturate GP activity. Cumene hydroperoxide concentration is also increased from 12 mM up to 60 mM so that lipid peroxidation is increased and GP activity therefore is limited only by GP levels in RBC membrane preparations. In each measurement of the enzyme activity the decrease of A_366 _was determined over a 2 min period. GP activity was standardised against Hb concentrations and expressed as NADPH mmol oxidized per minute per mg of hemoglobin (mmol/min/mg Hb). All chemicals were from Sigma Aldrich (St. Louis, MO).

## Results

**Figure 4:**
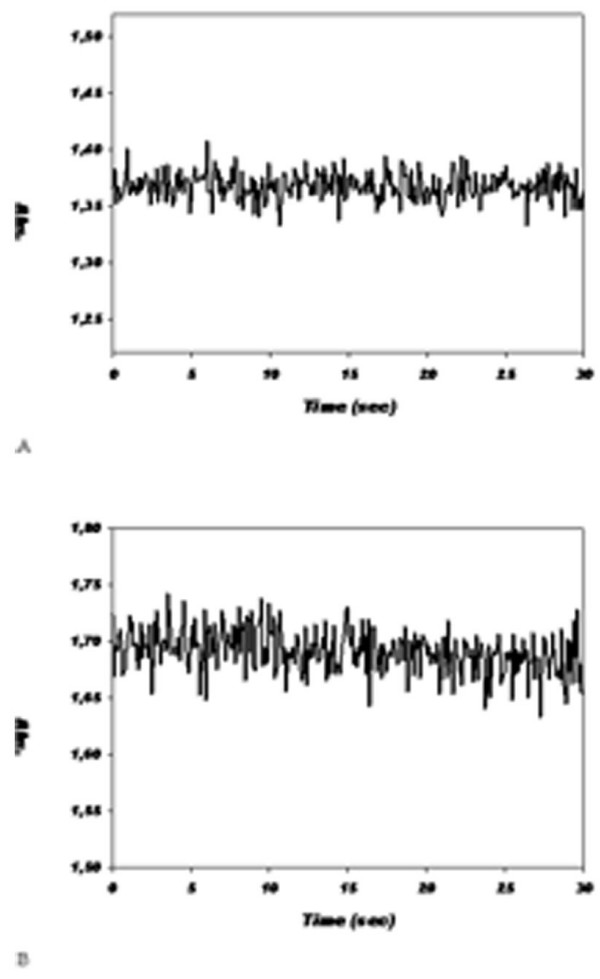
**Effect of optimization of the buffer composition and enzyme substrates and cofactors concentrations**. A according to the method of Paglia and Valentine. B after optimization of substrate and cofactor concentrations by experimental evaluation of the GP activity of hemolized rat RBC at 37°C.

**Figure 5:**
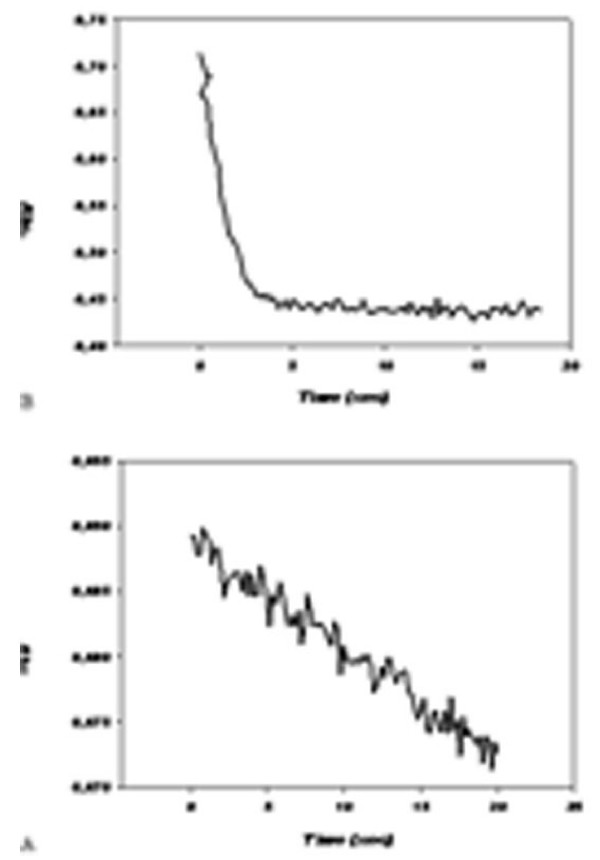
**Comparison of typical GP kinetics of pregnant (A) and nulligravid (B) RBC GP activity at 37°C**.

The GP activity is studied by spectrophotometrical analysis at least long lasting 20 s at 37°C, pH 7.0, wavelength 366 nm. In a first series of experiments, GP was determined at several final concentrations of GSH, and successively of NADPH, of GR and of cumene hydroperoxide in the described order. In Fig. 4A The time course of the GP reaction according to Paglia and Valentine method prescriptions is described. In Fig. 4B is described a typical time course obtained after the optimization of previously cited reaction parameters. As evident in fig. 4B the absorbance of NADPH decreases linearly after at least for 20 s of observation in contrast to an almost constant absorbance of Fig. 4A. Thus does not allow to calculate an appreciable speed of NADPH oxidation of the cited reaction. In Fig. 5A is described the kinetics of GP activity of RBCs from 140 d old pregnant female rats at 18 d of pregnancy. The kinetics is slow and does not reach equilibrium within 20 s. In Fig. 5B is described the kinetics of GP activity of RBCs from 140 d nulligravid female rats. The reaction, in the conditions described in table 1, has a high V_i _and is almost at equilibrium within 5 s. As described in table 2 the V_i _in normal rats is about 135.30 10^-6 ^mol/s and 3.96 10^-6 ^mol/s in pregnant rats, that is one fiftieth of the reference activity.

**Table 1:** Optimal concentrations of Glutathione peroxidase reaction medium.

Content	Concentration
Potassium phosphate, containing 2,5 mM Na_2_EDTA, 2,5 mM sodium azide, pH 7.0	0,5 M

Glutathione reductase (from baker yeast, ammonium sulfate suspension, 100–300 U/mg protein) in the same phosphate buffer;	0,18 U/ml: at 25°C

GSH (>98%) in distilled water	100 mM

NADPH (~95%) in 0,1% NaHCO_3 _solution	10 mM

Cumene hydroperoxide (>80%) in distilled water	60 mM

**Table 2:** Initial velocity of GPX complex activity in normal and gravid RBC membranes of 140 days old female rats (mean ± S.E. M. of eight indipendent experiments)

Vi (moles/sec) normal	Vi (moles/sec) gravid
135,30 10^-6 ^± 1,72 10^-6^	3,96 10^-6 ^± 0,83 10^-6^

## Discussion

**Figure 6:**
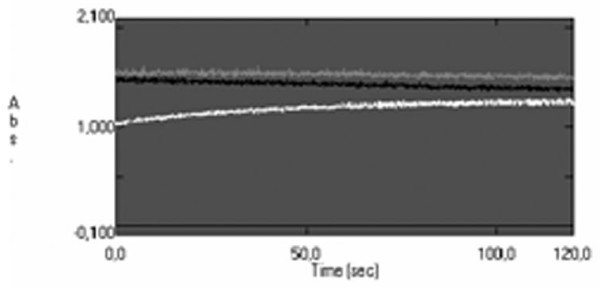
**GP activity as function [GSH]**. Each point is the mean of three independent determinations.

The described revision of Paglia and Valentine [1] method to evaluate GP activity in RBCs membranes of mammalia must be optimized for the specific kind of samples studied. The modifications shall be devolved to obtain the stable function of studied enzyme activity under saturation conditions [15] Only in this instance the time course of absorbance of NADPH at 366 nm describes the real kinetic behaviour of GP catalized redox reaction. The principle has been applied by optimizing the concentrations of substrates and cofactors of both GR and GP activities in order to obtain that only GP is the limiting enzyme activity of our RBC enzyme complex system. GP-Activity and kinetics were measured at 13 mM, 10 mM e 7 mM GSH. (figure 6). The striking feature is that the timecourses are useful to screen between the three GSH concentrations. By using the scientific software SigmaPlot (derivative), Enzyme Kinetics, (kinetics) subroutine, and the statistic analysis on SigmaStat. To shorten the description, the use of NADPH at several concentrations is not described here, but was performed by the same statistical methods of analysis previously described for GP. Special attention should be applied to cumene peroxide concentration optimization, according to the short term stability of this reactant in laboratory environment conditions. Also the RBC membrane preparations show short term stability if not stored at 4°C and utilized within 24 hours. The NADPH absorbance data at 366 nm are interpolated according to Hofstee and Eadie [8,9] by derivative routine of SigmaPlot^® ^scientific graph program. The optimized method was therefore applied to investigate in rats the effect of pregnancy on oxidative stress action in RBC membranes as described by GP activity evaluation. As the GP activity was previously studied only in tissues such as rat liver and placenta without optimization of substrata conditions [7], the selection of blood as the target compartment to study the effect of systemic peroxide levels has the advantage of investigating the compartment subjected to all the comprehensive effects of peroxide systemic accumulation. In fact, data show the significant increase of GP activity during pregnancy in comparison to normal age paired nulligravid female rats. Such a clearcut increase confirms the working hypothesis that the adaptative reaction to oxidative stress in pregnancy includes the increase of GP enzyme antioxidant activity. In figures 5 and 6 the significance limit is set at *p *< 0.05 of results and is accomplished by two ways Student t, using SigmaStat^® ^scientific statistical program.

## Conclusion

The described results show that, after the optimization of enzyme kinetics measurement conditions, the GP activity of pregnant rats is clearly distinct by the GP activity of female age paired nulligravid animals.

This indeed is well in agreement with the metabolic increased oxidative metabolism of pregnant animals [16]. Moreover the hormonal rearrangement intrinsic of pregnancy [17] redistributes the interrelationships of metabolic pathways that sustain fetus growth and differentiation, particularly in rats, that have a short pregnancy (about 17 d).

## Competing interests

The authors declare that they have no competing interests.

## Authors' contributions

GG carried out GP method optimization and evaluation of GP activity in pregnancy. GM, director of research, carried out control of animal fertility, both kinetic and statistical analysis of spectroscopic data. All authors read and approved the final manuscript.
